# A controlled trial of implementing a complex mental health intervention for carers of vulnerable young people living in out-of-home care: the ripple project

**DOI:** 10.1186/s12888-016-1145-6

**Published:** 2016-12-07

**Authors:** Helen Herrman, Cathy Humphreys, Stephen Halperin, Katherine Monson, Carol Harvey, Cathrine Mihalopoulos, Susan Cotton, Penelope Mitchell, Tony Glynn, Anne Magnus, Lenice Murray, Josef Szwarc, Elise Davis, Sophie Havighurst, Patrick McGorry, Sam Tyano, Ida Kaplan, Simon Rice, Kristen Moeller-Saxone

**Affiliations:** 1 Centre for Youth Mental Health, The University of Melbourne, 35 Poplar Road, Parkville, VIC 3052 Australia; 2 Orygen The National Centre of Excellence in Youth Mental Health, 35 Poplar Road, Parkville, VIC 3052 Australia; 3 Department of Social Work, University of Melbourne, Parkville, VIC 3010 Australia; 4 Orygen Youth Health, 35 Poplar Road, Parkville, VIC 3052 Australia; 5 Psychosocial Research Centre Department of Psychiatry, University of Melbourne, 130 Bell Street, Coburg, VIC 3058 Australia; 6 North Western Mental Health, Melbourne, Australia; 7 Centre for Population Health Research, Faculty of Health, Deakin University, 221 Burwood Hwy, Burwood, VIC 3125 Australia; 8 Youth Support and Advocacy Service (YSAS), 131 Johnston Street, Abbotsford, VIC 3065 Australia; 9 Integrated Mental Health Program, Royal Children’s Hospital, 117-129 Warringa Cres, Hoppers Crossing, VIC 3029 Australia; 10 Foundation House - Victorian Foundation for the Survivors of Torture Inc, 4 Gardiner Street, Brunswick, VIC 3056 Australia; 11 The Jack Brockhoff Child Health and Wellbeing Program, Centre for Health Equity, The Melbourne School of Population and Global Health, The University of Melbourne, Level 5, 207 Bouverie St, Melbourne, VIC 3010 Australia; 12 Department of Psychiatry, Mindful, Centre for Training and Research in Developmental Health, University of Melbourne, Building C, 50 Flemington Street, Flemington, 3031 Australia; 13 Sackler School of Medicine, Tel Aviv University, Ramat Aviv, Tel Aviv, Israel

**Keywords:** Out-of-home care, Youth mental health, Prevention, Implementation, Complex intervention, Looked after children

## Abstract

**Background:**

Out-of-home care (OoHC) refers to young people removed from their families by the state because of abuse, neglect or other adversities. Many of the young people experience poor mental health and social function before, during and after leaving care. Rigorously evaluated interventions are urgently required.

This publication describes the protocol for the Ripple project and notes early findings from a controlled trial demonstrating the feasibility of the work. The Ripple project is implementing and evaluating a complex mental health intervention that aims to strengthen the therapeutic capacities of carers and case managers of young people (12-17 years) in OoHC.

**Methods:**

The study is conducted in partnership with mental health, substance abuse and social services in Melbourne, with young people as participants. It has three parts:

1. Needs assessment and implementation of a complex mental health intervention; 2. A 3-year controlled trial of the mental health, social and economic outcomes; and 3. Nested process evaluation of the intervention.

**Results:**

Early findings characterising the young people, their carers and case managers and implementing the intervention are available. The trial Wave 1 includes interviews with 176 young people, 52% of those eligible in the study population, 104 carers and 79 case managers.

**Conclusions:**

Implementing and researching an affordable service system intervention appears feasible and likely to be applicable in other places and countries. Success of the intervention will potentially contribute to reducing mental ill-health among these young people, including suicide attempts, self-harm and substance abuse, as well as reducing homelessness, social isolation and contact with the criminal justice system.

**Trial registration:**

Australian New Zealand Clinical Trials Registry ACTRN12615000501549. Retrospectively registered 19 May 2015.

## Background

More than eight million young people worldwide who have been abandoned, orphaned, neglected or are otherwise unsafe at home live in large institutions or orphanages, under adverse conditions including strict routines, lack of personal relationships and isolation from wider society [1]. Many countries promote foster-care programs as a solution to better care for these vulnerable children. Foster care programs need to be well designed and support stable placements, given unstable placements are likely to be as harmful as institutionalisation to a child’s future mental health and function [1].

In Australia, foster care and similar programs are known as ‘out of home care’ (OoHC). OoHC is provided to young people up to 18 years old who are removed from home by the state because of significant risk of harm from abuse, neglect or other adversity. Many have multiple and complex needs, with poor mental health and social function before, during and after care [2, 3].

### Young people in OoHC

The three main types of OoHC are: foster care, in the private home of a substitute family receiving payment for the child's living expenses; kinship care, with a family member or approved custodian (both constitute home-based care); and residential care, in a house with up to four young people supported by paid staff [2]. Each week in the Australian state of Victoria (population 6 million) over 60 young people are placed in OoHC. On any single day approximately 7700 are in OoHC placements, about one-third of whom are aged 12-17 years [4]. Around 90% live in home-based care and the remainder in residential care [2].

The number of children and young people in OoHC in Australia is escalating, as more stay longer in care [4]. In 2013-14, there were 43,000 living in OoHC, more than double the national numbers in 2001 [4]. Aboriginal and Torres Strait Islander (ATSI) young people are living in OoHC at almost ten times the rate of their non-ATSI counterparts [4]. The Australian Productivity Commission estimated direct national expenditure on child protection services in 2009-10 as $2.5 billion dollars, of which OoHC accounted for 65% or $1.7 billion [5]. A similar situation exists in other countries including the USA [6], and the UK [7].

### Problems with mental health and social function in OoHC recipients

The risk of mental ill-health for young people living in OoHC is notably higher than in the general population. There are high rates of emotional and behavioural disorders among children in foster care in the USA, UK and Denmark, as determined on rating scales such as the Child Behaviour Checklist and Strengths and Difficulties Questionnaire (SDQ) [8–10]. In an English national prevalence study, [9] 45% of ‘looked-after children’ had a diagnosable mental disorder, compared with 10% in the general population. Those with mental health problems and disorders were also more likely to have educational, health and social problems [9, 11]. As well as disrupted families and exposure to emotional, physical and sexual trauma, poverty and other adversities, young people in OoHC commonly experience problems with alcohol and other drugs, suicidal ideation and self-harm, delinquency, and truancy [12–14]. These findings are consistent across studies in Australia, USA, UK and Denmark [8–10, 12–14]. The young people are also less likely to have timely access to mental health care [9, 15–17]. Proactive, regular and voluntary help-seeking is infrequent among vulnerable young people [17, 18].

Young people are legally required to leave the state protection of OoHC at the age of 18 in Australia. They then encounter limited opportunities for work or further education and are at significant risk of homelessness [3]. A longitudinal study of young people leaving care in Australia reported that nearly 50% had attempted suicide within four years [19]. One in three young women had become pregnant or given birth within 12 months of leaving care [12]. Thirty five percent of young people in state care in another study had become homeless within 12 months of leaving care [20].

International research confirms that foster care children who experience placement disruption and instability are at heightened risk of a range of poor outcomes [21]. Young people in Australia who were settled in one placement for at least 75% of their time in care had better outcomes a year later than those with multiple placements [12].

### Therapeutic mental health care in the OoHC system

The OoHC system needs to be based on therapeutic care principles. A therapeutic response is defined as: appropriate to the background of abuse and neglect and the problems related to emotions, behaviour and functioning common to many young people in the sector. All forms of OoHC risk re-traumatising young people by failing to respond to their needs [2, 22–25]. Improvements in mental health services are unlikely to have much effect unless OoHC systems become more therapeutic [23]; and simply providing day-to-day care for children and young people in OoHC is no longer an adequate approach [2, 24]. A recent international review identified one intervention that met review criteria for adolescents in OoHC, the Multidimensional Treatment Foster Care for Adolescents (MTFC-A; [26]). MTFC trains foster carers to deal with young people with high behavioural and mental health needs enabling those young people to stay out of residential care and receive individual, intensive support [27]. Carers are supported by a team of professionals who aim to work proactively with the young person and prevent behavioural and mental health problems and subsequent placement breakdowns. The review of MTFC-A described the existing evidence for its effectiveness as weak, and recommended that rigorous evaluation studies, while difficult to conduct, are sorely needed in this field [28].

Australia’s National Standards for OoHC [29] recommend that children and young people have their physical, developmental, psychosocial and mental health needs assessed and addressed in a timely way. Cross-service practice models offer integrated and trauma-informed mental health support in Victoria and elsewhere [22, 30]. These reach approximately 10% of all young people in OoHC in Victoria. There is evidence that cross-service models can be successfully implemented [31] and are associated with improved placement stability for adolescents [32]. However, child and youth services generally have tenuous links with health services and have been slow to implement evidence-based practices in mental health [33, 34]. These young people require innovative approaches to delivering evidence-based mental health practice, including cross-sector collaboration. The common approaches do not reach most of them nor provide appropriate responses to their needs [34].

Australian National Standards for OoHC [29] note the importance of maintaining cultural links for the young people, including Aboriginal and Torres Strait Islander (ATSI) young people. There is little information in Australia about young people in OoHC either from ATSI or other culturally and linguistically diverse (CALD) groups, including those with refugee backgrounds [2, 35] and associated experiences of trauma, dislocation and loss [2].

### Carers supporting young people’

The environment and individual relationships with the young person are both crucial in the therapeutic and recovery process [20]. Those caring for young people need an understanding of trauma and its impact and the capacity to respond in an attuned way to promote mental health [20, 30]. Most carers are helped by basic theoretical knowledge, familiarity with evidence-based techniques, and a trusted adviser with whom to discuss instances of disturbed behaviour and emotional responses. Specific techniques can help the young person learn to regulate emotions, accept painful feelings, promote direct expression of feelings appropriately rather than withdrawing or behaving destructively, desensitise traumatic memories and promote unified thoughts, feelings and behaviours [20].

There are calls for greater attention to the training and support needs of carers, their wellbeing and retention to caring [36, 37]. Many carers report high levels of stress and poor coping [36–38]. Irrespective of their capacity to identify mental ill-health, many do not gain access to mental health care for the young person [39, 40]. When they do have access, carers often feel excluded from treatment planning and implementation [41]. Mental health training and consultation improve carer wellbeing, and increase the likelihood of access, when warranted, to mental health services [42, 43].

### Working in collaboration across the service systems

Collaboration is essential for tackling complex problems with roots in multiple sectors of society [44]. Poor mental health among young people in OoHC is one such complex problem. Tackling this problem requires collaboration between mental health and social services, and is most likely to succeed when the partners are clear about their respective roles [45, 46] and understand the differences in their viewpoints about therapeutic approaches for young people with multiple and complex needs [18, 22]. Australian studies suggest that a prominent barrier to collaboration in OoHC is frustration among professionals related to a lack of common understanding of the young person’s situation arising from differing professional frameworks for addressing problem behaviours. Other barriers to collaboration include power imbalances within and between professional groups, along with insufficient resources to actually address problems [47].

### Implementing evidence-based mental health practice – EBP – for vulnerable young people

Several different therapeutic approaches are effective in reducing emotional and behavioural problems and improving mental health for adolescents with multiple and complex needs [48, 49]. These include: motivational interviewing (MI), cognitive behaviour therapy (CBT), and adolescent community reinforcement approach (ACRA) [49]. A modular practice elements approach to selecting and designing EBP (‘EBP elements’) is a flexible option for young people with multiple and complex needs [34, 48–50]. Mental health services in a variety of locations and contexts have adopted this approach effectively [51, 52]. Common problems for young people in OoHC and amenable to treatment through EBP elements include: (i) emotional dysregulation (manifesting as anger, deliberate self-harm, and suicidal ideation); (ii) insecure attachment (manifesting as difficulties forming stable emotional bonds with others); (iii) low self-efficacy and expectations for the future; and (iv) limited social problem-solving skills [48–50].

A complementary approach to addressing the needs of young people is training for carers in emotional responsiveness. The Tuning into Teens (TINT) program [53] is an emotion-coaching program for parents that reduces emotional and behavioural problems in young people. The TINT program has been adapted for use in OoHC.

### Implementing and evaluating an innovative mental health intervention

We found no studies of a systematic approach to improving mental health for adolescents in ‘usual care’ OoHC in Australia. US and UK studies focus on the complexity of integrating EBP into therapeutic foster care programs run by child welfare agencies [54, 55]. Implementation science indicates that the changes needed to embed evidence-informed practice into complex systems occur in stages [56]. The first or exploration stage involves sustained work to establish readiness for change. Interested parties engage in mobilising support, establishing feasibility and developing commitment to the innovations [57]. The Ripple study design reflects the understanding that when shifting practice to focus on improving mental health of young people in OoHC, this early stage in implementation is critical.

### The Ripple project

The project concerns young people in Melbourne (capital city of the Australian state of Victoria) living in all types of statutory OoHC. The Victorian government’s department of Children, Youth and Family Service contracts with community service organisations (CSOs) to provide OoHC. The project investigates the implementation of an innovative mental health intervention in OoHC settings managed by CSOs. A recent mental health policy initiative in Victoria prescribes priority access to state-funded mental health services for young people in OoHC and emphasises support for carers and case managers [58]. Design of the Ripple intervention draws on this and other observations: improving coordination between mental health and OoHC services and increasing capacities of staff and carers in both sectors to work together are prerequisites to improving mental health and social functioning for vulnerable young people [45].

The intervention is described in Table 1. Its features include collaboration between OoHC and mental health organisations and tailored delivery of. evidence-based mental health support using skilled mental health and alcohol and other drug (AOD) knowledge and skills. Senior mental health or AOD practitioners schedule regular visits (2-4weekly) to CSO program sites. Practitioners are trained and supervised in using community development and adult-learning principles to develop one or more six-session intervention plans collaboratively with CSO workers. All CSO programs are offered a choice in delivery modes (such as practice group discussions - See Table 1 - “modes”) and topics (see Table 1 - “content section”). The tailored approach to planning the mode and content of the intervention is responsive to the learning styles and interests of each CSO program, and it allows for changes based on the groups’ evolving needs and experience of the intervention. Participation of young people with experience of OoHC is likely to improve the chances of a successful intervention by creating a positive climate, generating useful ideas and links to decision makers. This is likely to improve the young peoples’ engagement, health and social outcomes and the quality of services and professional work [59–62].

**Table 1:** Overview of the Ripple mental health intervention: a complex mental health intervention in Melbourne’s North West Metropolitan Health Region (NWMR)

Main components	Activities	Description
A. Organisational Collaboration and Contribution Developing shared language and commitment Fundamental to the mode and content of delivery of the intervention	Implementation Group established at each Community Service Organisation (CSO)	Lead Ripple practitioners, Ripple project staff, and CSO management and staff meet regularly to review activity and opportunities and adapt the intervention as required
	Ripple Practitioner Training and Supervision	Skilled mental health and alcohol and other drug (AOD) practitioners selected from 3 partner mental health agencies, trained and supervised (by lead practitioners from the same agencies) to work part-time with the CSOs
	Youth Peer Leaders	Young people with lived experience co-deliver training and other selected activities
B. Mode of Delivery Ripple practitioner with specialist mental health expertise visits regularly A Ripple practitioner visits an identified CSO worksite^a^ at specified times: to develop relationships, establish trust, and strengthen CSO staff capacity to use mental health concepts and skills in their work	Professional Practice Groups	Ripple practitioner and CSO staff meet regularly for reflective practice; skills training; and case discussion
	Training Sessions	CSO staff nominate key areas or topics for training on key mental health or AOD topics
	Secondary Consultation and Care Team Consultation	Ripple practitioner available by special arrangement for staff or care-team consultation about a specific young person
	Carer Groups Training in emotional attunement, psychological skills	CSO staff trained in TinT: option to train carers in TinT courses
C. Content Mental health/ Alcohol and drug expertise and evidence-based knowledge	Support for skills training and reflection for CSO staff with use of Toolbox and TinT experience	CSO staff trained in i) mental health & AOD signs, symptoms, and support strategies, ii)accessing and navigating mental health support iii) psychoeducation and evidence based support strategies such as motivational interviewing techniques and emotion-focused coaching
Tuning into Teens (TinT) Training program for carers and parents of teenagers (see Reference 52)	Selected CSO staff trained as TinT trainers for: carer groups; and CSO staff	TinT adapted for OoHC to support the development of emotional attunement and emotion-coaching roles for those caring for adolescents. TinT program staff provide training and supervision
Toolbox http://www.youthaodtoolbox.org/ http://oohc.webtribe.com.au/	Online information on evidence-based therapeutic practice elements	Toolbox adapted for use in OoHC. Ripple practitioners trained to support CSO staff in its use
D. Menu of Therapeutic and Mental Health Knowledge and Skills^b^	Challenging behaviours (and the role of trauma in their development and maintenance); Emotion regulation; Depression and anxiety; Communication and social skills; Family-focused interventions; Motivational interviewing; Problem solving; Promoting resilience; Self-harm and suicide	

^a^Each CSO has several worksites for home-based care teams and residential care houses

^b^Training, reflection and supervision available to CSO staff in this range of skills, used according to the agenda chosen at each worksite

The four Community Sector Organisations (CSOs) involved in the project are large, multi-functional, not for profit organisations. The Victorian Aboriginal Child Care Agency (VACCA) has a focus on Aboriginal families. Like the other organisations (Anglicare Victoria, Mackillop and Westcare), they cross the spectrum of programs to respond to vulnerable families providing early intervention, intensive family support services, and services for children, families and carers in out of home care. The staff in all organisations include highly qualified social workers and psychologists, as well as those with baseline qualifications at Certificate level. Aside from VACCA, the organisations hold strong religious affiliations though the employment of staff is not based on professional rather than religious affiliation. Most of the programs the CSOs provide are funded by government.

### AIMS

The paper aims to describe the protocol for the Ripple project and to note early findings from a controlled trial demonstrating the feasibility of the work. The Ripple project is implementing and evaluating the complex mental health intervention described above that aims to strengthen the therapeutic capacities of carers and case managers of young people (12-17 years) in OoHC.

The overall aims of the Ripple project are to assess whether a mental health intervention that enhances therapeutic care roles and capacities of carers in OoHC will improve (i) consistency and quality of OoHC for all young people (12-17 years) in the sector, and (ii) access to early intervention, when indicated, for prevention and treatment of mental illness in a cost-effective manner. Both outcomes will likely contribute to improving the mental health of young people in OoHC.

### Primary hypothesis

After three years, young people in OoHC in a region in which the intervention is delivered (intervention group) will have better mental health, and better social functioning than those receiving care as usual (comparison group), on the outcome measures of i) emotional distress, harmful use of substances, and disturbed conduct; and ii) social relationships, education and occupation.

### Secondary hypotheses

In the intervention group compared with the comparison group, after three years: (i) young people in OoHC will receive more evidence-based psychosocial interventions, have fewer placement changes, and report more community connections and greater sense of empowerment; (ii) foster and kinship carers and residential care workers (carers), and child protection and CSO workers (case managers) will have greater satisfaction with their work, less stress, improved health-related quality of life, improved attitudes towards EBP and provide a higher quality care environment; (iii) services will operate with more effective collaborative arrangements; and (iv) the intervention will be a more cost-effective approach than treatment as usual to improving the mental health of young people in OoHC.

## Methods

The Ripple project has several components: *1) Needs assessment and implementation of a complex mental health intervention; 2) Controlled intervention trial to determine the impact on mental health, social and economic outcomes; and 3) Nested process evaluation of the intervention.*

**Fig. 1:**
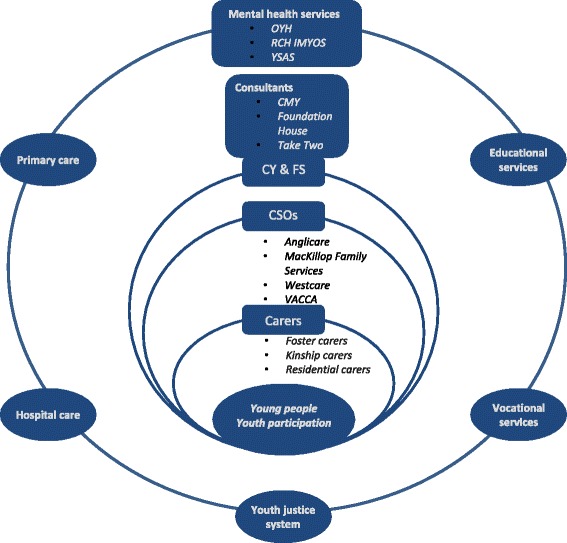
Health and Community Service System for youth in OoHC in Victoria^1^. Legend - OYH: Orygen Youth Health Clinical Program; RCH IMYOS: Royal Children’s Hospital Intensive Mental Health Youth Outreach Service; YSAS: Youth Support and Advocacy Service; CMY: Centre for Multicultural Youth; CY & FS: Child, Youth and Family Services; CSOs: Community Support Organisations; VACCA: Victorian Aboriginal Child Care Agency. ^1^Specialist mental health services for young people aged 12-17 years in Victoria are provided by community-based state-funded mental health services [18]. Their contact with young people in OoHC and with the CSOs is typically ad hoc rather than organised systematically. Foundation House (the Victorian Foundation for Survivors of Torture and Trauma), the Centre for Multicultural Youth (CMY) and Take Two (state-provided psychological services for children and young people in OoHC) provide services relevant to some or all of these young people. They are consulting study partners. These organisations all interact with primary health care, hospital emergency and inpatient services, police, justice, education and vocational services. The Ripple intervention region is Melbourne’s North and West Metropolitan Health Region (NWMR). NMWR has a culturally diverse population of 1.7 million people, including 24% of Victoria’s Aboriginal people (0.58% of the region’s population). It comprises Melbourne’s central business district and inner, middle and outer urban areas. OYH, RCH IMYOS, and YSAS provide specialist mental health services for young people in this region. Four major CSOs in this region are study partners: Anglicare, MacKillop Family Services, Westcare and VACCA

The project is conducted with four major CSOs in Melbourne: Anglicare, MacKillop Family Services, the Victorian Aboriginal Child Care Agency (VACCA) and Westcare (Salvation Army). A complex health and community service system in Victoria surrounds CSOs and the young people in OoHC (see Fig. 1). Specialist mental health services are provided by community-based state-funded mental health services [18]. Their contact with young people in OoHC is typically ad hoc rather than organised systematically. Foundation House (the Victorian Foundation for Survivors of Torture and Trauma), the Centre for Multicultural Youth (CMY) and Take Two (state-provided psychological services for children and young people in OoHC) provide services relevant to some or all of these young people and are consulting study partners. These organisations interact with primary health care, hospital emergency and inpatient services, police, justice, educational and vocational services.

The intervention region is Melbourne’s North and West Metropolitan Health Region (NWMR). NMWR has a culturally diverse population of 1.7 million people, including 24% of Victoria’s Aboriginal people (0.58% of the region’s population). It comprises Melbourne’s central business district and inner, middle and outer urban areas. Specialist mental health services for young people aged 12-17 are provided by Orygen Youth Health (OYH), the Royal Children’s Hospital (RCH), and the Youth Support and Advocacy Service (YSAS). The four CSOs each provide OoHC in the south or east regions of metropolitan Melbourne that comprise the region from which the comparison group is drawn. Youth mental health services not linked to the study are responsible for mental health service delivery in these regions.

Youth and Carer Participation. The research team is collaborating with the CREATE Foundation office in Victoria (www.create.org.au; a national not-for-profit organisation representing the interests of young people in OoHC) to train and engage young people in the study in roles including co-delivery of training sessions, participating in research project meetings and co-delivery of research presentations. Young people are involved in all three study components. Carers have been recruited into a nested qualitative study to comment on current training and support and recommend improvements.

### Research plan

- *1. Needs assessment and implementation of a complex mental health intervention.*
  Before implementation of the intervention in NWMR, young people, carers and case managers participated in focus groups and interviews to assess mental health needs and experiences of service delivery. Mental health services, child protection services, CSOs and advocacy organisations consulted with researchers about service delivery, policy and training in OoHC and mental health services. Three meetings of stakeholders were convened. Aboriginal and Torres Strait Islander and culturally and linguistically diverse young people contributed to additional studies of their needs.
  The intervention design is informed by this assessment of needs. The intervention aims to work with CSO case managers to support carers of the young people aged 12-17 living in OOHC in the intervention region and thereby: promote mental health and prevent mental ill-health and associated behavioural problems for the young people; and encourage early access for the young people, when needed, for treatment and support for mental illnesses and behavioural problems. Ripple practitioners and trained young leaders conduct the intervention. The Ripple practitioners are experienced youth mental health clinicians or AOD workers, seconded to work part-time with the study partners following a process of internal recruitment, and are subsequently trained and supervised by senior colleagues. The young leaders are aged 18-25 years, have experience in OoHC and are trained in leadership in mental health in a parallel study conducted in association with CREATE (the Bounce study).
  The intervention is implemented in collaboration with the CSOs and statutory child protection services. It provides flexible delivery of evidence-based, trauma-informed reflection, education and training for case managers in home-based and residential care teams in the CSOs. It includes training and support for case managers in the use of EBP elements, in emotional responsiveness, and in practical ways to support carers in: responding therapeutically to young people in their care; and gaining appropriate access to primary health care and specialist mental health support. The involvement of educational and vocational services is promoted. More effective collaborative arrangements are operationalized through the tailored component of the intervention design (see Table 2). That is, by tailoring the mode and delivery of the intervention to the individual needs of services, more effective collaboration between mental health services and CSOs should result.

**Table 2:** Ripple study measures and procedures

Outcomes for young people	Measures
Enhanced social and emotional wellbeing	Strengths and Difficulties Questionnaire (SDQ)[67], empowerment graphic (S. Davidson, personal communication)
Enhanced quality of life	Child Health Utility Index (CHU9D)[87]
Better engagement with school	Selected questions from Pathways Longitudinal Study [70], Beyond 18 Study [71]
Placement stability and experiences of contact with family of origin	Selected questions from Beyond 18 Study [71], demographic questions
Fewer mental health problems	K10 [66]
Less harmful use of substances	ASSIST-Y [68]
Improved social function	Friendship Scale [69], SDQ [67] peer & prosocial subscales
Less involvement in crime	Questions developed by study team for economic evaluation - Resource Use Questionnaire
Outcomes for carers and case managers	
Enhanced health-related quality of life	AQoL8D [88]
Increased satisfaction, sense of competence and efficacy with carer role; better access to self-care and support	Carer Users Expectations Survey (CUES) [89]
Better relationships with young people	Family Assessment Device – General Functioning Scale (FAD-GFS) [90]
Improved skills for managing behaviours and emotions of concern	Emotions as a Child questionnaire (EAC) [91]
Outcomes for services	
Measure of organisational learning capacity and readiness for transformational change in human services	Organisational Learning Capacity Scale (OLCS) [92]
Costs and cost offsets of the Ripple intervention	
The costs of the Ripple intervention The costs are calculated to include the time commitment of the Ripple practitioners, valued at professional award rates, and the recipients of their reflective, educative and consultative professional services.	Multiple data sources, including: a. Time-use records maintained by Ripple practitioners b. Information from youth, carer and case manager respondents c. Administrative data sets and interviews with key budgetary personnel
Cost offsets of the Ripple intervention - Broad resource use of participating young people and carers	The Resource Use Questionnaire developed for the project collects broad resource use of participating young people and carers over the previous year for both assessment waves: including use of health and welfare services, educational attendance and workforce productivity and contacts with civic compliance authorities. Carers provide relevant information about themselves and the consenting youth in their care. All participating young people and their carers are asked to consent separately to the release of their confidential medical and pharmaceutical service use history (an administrative dataset) for the preceding 12 months, from the Australian Government.
The costs of consulting health professionals	Valued using published prices available in Medicare and Pharmaceutical Benefits Schedules.
Costs to the education and justice sectors	Derived (top down) from publicly available cost data.
Interview and data management procedures for both waves: A CSO case manager contacts each young person and carer to ask permission for contact from a trained Ripple research assistant. At a face-to-face meeting in their preferred location, the respondents are asked for informed consent and assured of privacy and lack of coercion before proceeding with the interview. Procedures for data management ensure anonymous data storage as well as appropriate linkage between each young person, carer and case manager. The measures are incorporated in an interview schedule that is administered face-to-face by trained research assistants. Respondents are invited to complete self-report measures on iPads provided. Research assistants provide help if requested by the participant, or otherwise assist with completion on paper. Data entry is automated for measures included on the iPads and otherwise completed and checked by the research assistants. Youth and carer participants are offered reimbursement of $30 for participation
  The agencies in comparison regions are provided with descriptive information about the characteristics of young people in their care following baseline study assessments (see below). This includes information about the mental health and function of the young people in their care. At the end of the study all components of the research intervention (see Table 1) will be freely shared with all involved.

**Fig. 2:**
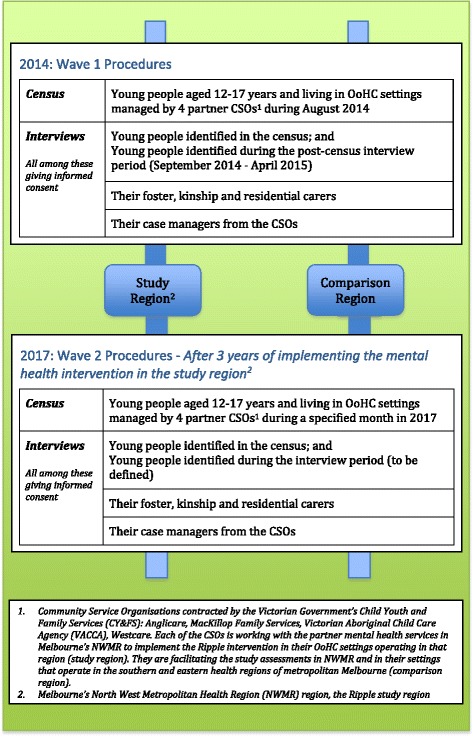
Design of the Ripple controlled trial of implementing a complex mental health intervention in out-of-home care (OoHC) settings in Melbourne
  Each assessment wave comprises: (i) A census of young people aged 12-17 years in OoHC settings managed by partner CSOs in intervention and comparison regions, to establish the study population at each time point; and (ii) interviews to collect information on the variables defined in the hypotheses using qualitative and quantitative measures conducted on: young people enumerated in the census and identified in the post-census period, the foster, kinship and residential carers and the case managers of these young people (see Fig. 2). Outcomes are measured on three levels: for young people in OoHC; their carers and case managers; and the services (see Table 2). The trial has been developed in accordance with the STROBE statement for cross-sectional studies [65]. According to EQUATOR, STROBE is the appropriate guideline for observational studies, including cross-sectional trials (see http://www.equator-network.org/reporting-guidelines/strobe/)."

- *2. A controlled trial of mental health, social and economic outcomes of the intervention*
  The trial is designed to answer the question: What is the effectiveness and cost-effectiveness of the Ripple mental health intervention for young people in OoHC, their carers, case managers and the CSOs compared with the current care situation? Outcomes of the intervention at individual and service levels are assessed through two waves of measurements in intervention and comparison regions; first at baseline as implementation begins, and then three years later (see Fig. 2). While there are challenges associated with this trial design, comparing local regions is the most pragmatic test of effectiveness in a real-world context, particularly if long-term or external influences on the settings are accounted for. [63, 64] A preliminary test of the comparability of the populations and settings is reported in the census data below.

### Primary outcomes

Primary outcome variables will assess changes to young people’s emotional distress (assessed by K10 [66]; SDQ Emotional Symptoms Subscale [67]), harmful use of substances (assessed by the ASSIST-Y [68]), disturbed conduct (assessed by the SDQ Conduct Problems & Hyperactivity subscales [67]), social relationships (assessed by the Friendship Scale [69], SDQ peer problems & prosocial subscales [67]) and education and occupation (assessed by measures adapted from the Pathways Longitudinal Study [70], Beyond 18 Study [71]).

Procedures: Approval for the trial was obtained from research ethics committees of The University of Melbourne (No. 1340674), Deakin University (No. 2014-046) and Anglicare Victoria (No. 2014-02) and ratified by research review committees of the other CSOs. The Victorian Department of Human Services Research Coordinating Committee approved the study. The Commonwealth Department of Health Australia (No. 14/2014) agreed to release Medicare and Pharmaceutical Benefits data for the economic evaluation (see below). Informed consent to participate in the study was obtained from participants, and in the case of children under 16 consent was obtained from their parents or legal guardian. The trial is registered with the Australian New Zealand Clinical Trials Registry (No. ACTRN12615000501549).

**Fig. 3:**
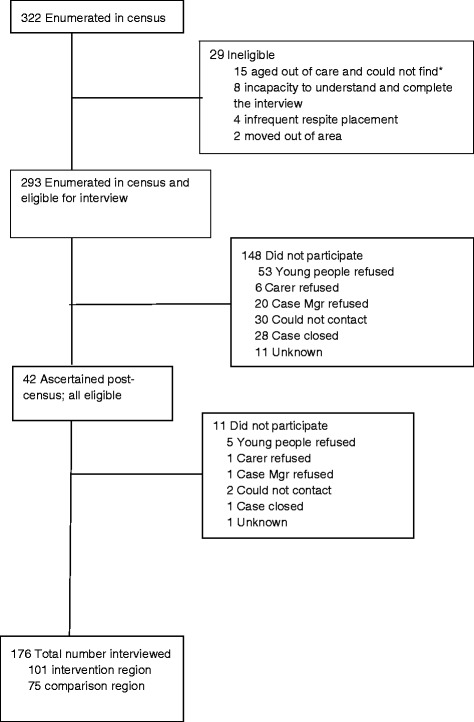
Ripple Project Interviews – Young People; Wave 1. *Note: Young people who were counted in the census but turned 18 during the interview period were deemed ineligible as they were out of age range and had left care. This had the additional effect of preventing follow up as agencies lose contact with young people at this age

Conduct of the census and recruitment of participants (See Fig. 3):

The Wave 1 census was conducted in August 2014. Research assistants visited CSO offices and supported by CSO staff completed a census questionnaire from file audit for each young person aged 12-17 in care during the designated two-week period. As many as possible of the young people identified in the census and eligible for interview, and their carers and case managers, were subsequently interviewed in Wave 1. Protocols were established for: (1) contacting and following up those enumerated in the census; and (2) risk management. Each young person and his or her carer or carers was contacted first by the young person’s case manager as identified by each agency. Recruitment was originally scheduled for completion in three months. In order to complete the agreed number of contacts and re-contacts for each potential participant to establish an appointment for the interview, the recruitment period extended to seven months. During this period an additional 41 young people who entered care during the recruitment period were identified and approached for interview using the same procedures.

### Statistical analysis

To determine group differences over time on outcome measures, a range of statistical techniques will be used: for categorical outcome measures, chi-square analysis (*χ*2), loglinear analysis, and logistic regression; and for continuous outcome measures, general linear models will be adopted. More sophisticated models will be considered to manage the hierarchical nature of the data obtained from the CSOs, case managers, carers and young people such as multilevel mixed (hierarchical) models.

Power calculation: During the study design, partner CSOs provided estimates of the numbers of young people aged 12-17 in OoHC settings managed by them in the intervention and comparison regions in any one month. Ultimately, the Wave 1 census revealed fewer young people in these settings than originally estimated. This meant that we did not need to select samples among the young people enumerated and their carers and case managers and instead aimed to interview all available. Given the lack of EBP research or sources of centralised information in OoHC, it is difficult to base power calculations on a priori measures of effect size.

### Economic evaluation design

Formal economic evaluation of the Ripple intervention compared with current practice will assess incremental cost-effectiveness from a societal perspective. This approach is useful to decision makers [72]. By including the health-related quality of life assessment of young people and their carers before and following the intervention, a cost-utility analysis can be undertaken also, allowing practical judgments about value for money of the intervention. The costs and cost offsets of the Ripple intervention are being measured using multiple data sources (see Table 2).

The economic analysis will include costs borne by several different sectors (health, education, justice). Secondary analyses can be undertaken from narrower perspectives, such as health or education, as relevant to the different stakeholders. If the Ripple intervention is found to be effective (in the short term, trial-based evaluation), the predicted future lifetime and wider population cost-effectiveness of the intervention will be determined using modelling techniques [73, 74].

3. *Process evaluation of the intervention* throughout the study is using multiple data sources and qualitative and quantitative data collection methods. Data is collected from all intervention sites in order to assess quality of implementation, clarify causal mechanisms and identify contextual factors associated with variation in outcomes [75]. Annual ‘Stocktake’ meetings and interviews with representative stakeholders from each CSO are conducted to assess implementation through documenting the activities, who carried them out, and the effort required [75]. The intervention team uses a participatory action research approach to continuous development of the method and content of their practice [76]. They engage in regular reviews at each intervention site through formal and informal verbal and written processes. Assessment of the effectiveness of collaborative arrangements is addressed through focus groups and a question in the OLCS.

## Results

### Progress and early results

The Wave 1 census identified the study population of young people for the first wave of assessments and provided information about culture, language and other variables among the young people. Nearly one in five young people enumerated in the census identified as Aboriginal or Torres Strait Islander. One in ten had a registered disability.

A total of 176 of 335 (52%) eligible young people identified in the census and during the interview period were interviewed (see Fig. 3). Interviews were obtained with 104 of 265 (39%) carers and 79 of 160 (49%) case managers, identified as linked with them.

Observations of implementing the intervention indicate that: (1) specialist mental health practitioners have been recruited and trained and their supervision established; and (2) their deployment to the OoHC settings is in progress and evolving in particular ways in different OOHC settings (see Table 1). ‘Stocktake’ meetings with each CSO have shared the results of the data collection, engaged CSO staff in the intervention and documented recommendations for improvement.

## Discussion

The Ripple intervention was originally conceived to strengthen the therapeutic capacities of the carers of young people in the Victorian OoHC system. This paper describes the intervention, co-designed with the participants and expert consultants from health and social service sectors, and early findings from Wave 1 of the controlled trial designed to evaluate the cost-effectiveness of the intervention. The context includes different practice models and approaches to mental health problems across the social service and mental health sectors [77, 78], and the limited mental health support for the carers and young people in OoHC [79]. Together these create areas in which there are broader implications for policy, practice and further research.

Preliminary observations from the implementation endorse the original broad conception for the intervention. The work to date suggests that it is feasible to implement a complex mental health intervention across sectors with relatively modest resources. The success of the intervention appears likely to depend on the strength of partnerships between organisations within and across service sectors. This is consistent with the emphasis in implementation science on ‘context’ [80, 81]. The implication of these findings attest to the policy imperative for a specialist mental health intervention of this type to support home-based and residential carers in their work with young people in care, and a readiness to work together to achieve this [80–82].

Early results from the field studies indicate that the research design can provide much needed information in this challenging field. Half of the eligible young people agreed to participate in the first wave of assessment interviews, despite coming from a vulnerable and hard to engage population, as did a third of their carers and half of their case managers. This rate of recruitment is considerably higher than that achieved in other studies of similar populations [83], implying that with appropriate resources and engagement at every level by the organisations involved, that quantitative research with this ‘hard to reach’ population is possible.

The census has been a useful source of information for partner CSOs, providing comparative data on the age, gender patterns and other characteristics of young people engaged by their services and the patterns of placement changes. These findings highlight the lack of routinely available information about the number, characteristics and circumstances of young people living in OoHC in Victoria. It is a situation that is reportedly similar in other Australian states and in other countries [84] and has significant implications for policy development to support more accurate and consistent data collection and analysis to inform practice in this area.

The census reveals 20 times more Aboriginal or Torres Strait Islander young people than their proportion in the population of Melbourne. This in part reflects involvement of VACCA as study partner. However 35% of identified Aboriginal or Torres Strait Islander young people were living in settings managed by other CSOs. There are profound implications for policy and practice to address this inequity. The recent report by the Aboriginal Children’s Commissioner points to some clear direction which include amongst other recommendations, more consistent and extensive exploration of safe placement within the extended family networks [85].

The reported rate of those with a registered disability (10%) among the young people enumerated in the census was lower than our original expectations. The expectations were based on impressions conveyed by CSO partners and other reports that 42% young people living in OOHC have a registered disability [86]. This discrepancy may reflect a lack of access to assessment and diagnosis of disabling conditions in community settings. It may also reflect the focus of the question on disability registered with state agencies, rather than the broader assessments including ‘suspected’ disability reported in other studies, a limitation with clear implications for future research in this area [86].

The early findings from participant interviews suggest that the data collection is acceptable and feasible. All young people, carers and case managers completed a minimum of demographic questions and the SDQ. We recruited lower than expected numbers into the study as a result of enumerating fewer young people in the census than originally estimated. This first interview wave represents, however, one of the largest groups of young people ever included in a mental health intervention study in OoHC, and Ripple is one of the few such studies conducted worldwide [55].

The higher estimates of sample size made initially with our partner CSOs reflect, at least in part, the limitations mentioned above in the routine information available to the CSOs. Calculations of the study’s power indicates that with the second cross-sectional wave of assessments we will be able to test main effects of the evaluation and will gain valuable information from economic and process evaluations. The limitations of the study provide directions for future research in the OoHC area. These limitations include the threat to generalisation of the results through the rate of recruitment from the study population, notwithstanding that the rate is relatively high for a study of this type as mentioned above. Analysing the characteristics of those recruited and not recruited will help us assess this risk. Another limitation is the focus on metropolitan youth in OoHC. Providing mental health services and OoHC in regional and rural settings is different in several respects from doing so in metropolitan areas (city and suburbs). The usefulness of these findings outside the metropolitan area cannot be assumed. However we anticipate that successful implementation will guide and encourage similar interventions and their evaluation elsewhere.

The design of the study followed the opportunities provided in the specific context of needs, political factors and organisational alliances and readiness in the authors’ setting. The concepts and processes used in this protocol are, however, potentially adaptable to other settings. They may be relevant in countries where resources are scarce and in any setting where efficiency of resource use is valued.

A feature of the controlled trial is its design with two cross-sectional waves. Although the absence of longitudinal data is a limitation of the study, the current design is a useful way to evaluate a complex intervention [63]. We anticipate that future studies will build on this foundation to develop cohort studies (to identify modifiable factors important to the mental health of young women and men of different ages) and more complex designs such as stepped-wedge cluster RCTs (to test the implementation and adaptation of mental health interventions in various types of OOHC and for specific groups of young people).

We predict in the meantime that the Ripple service model will result in case managers and carers in OoHC environments being more attuned to the emotional state and experiences of young people, and gaining better access to early intervention to prevent and treat mental illness. We can expect that both these changes will contribute to better mental health among young people in OoHC. We expect this will contribute in the longer term to improved quality of life and better productivity for the young people, and reduction among them in negative psychosocial outcomes.

## Conclusions

The central features of the Ripple intervention include youth participation and a modest allocation of specialist mental health staff to collaborate in providing a complex evidence-based mental health intervention. It responds to needs identified in a critical government inquiry in the Australian state of Victoria [2] similar to those identified in other states of Australia and in other countries. The approach is potentially adaptable to the needs elsewhere. In contrast to and complementary to other interventions trialled among vulnerable young people [22], it is a universal intervention that requires less intensive support from skilled mental health professionals. It is sensitive to context and culture and responds to the strengths of the young people and the adversities in their lives, using a service model that is designed to be sustainable and increase capacity across the broader service system. The evaluation of this intervention is continuing with the process evaluation and the second cross-sectional wave of assessments.

## Abbreviations

ACRA: Adolescent community reinforcement approach
ATSI: Aboriginal and Torres strait islander
CALD: Culturally and linguistically diverse
CBT: Cognitive behaviour therapy
CMY: Centre for multicultural youth
CSO: Community Service Organisations
EBP: Evidence-based mental health practice
MI: Motivational interviewing
MTFC-A: Multidimensional treatment foster care for adolescents
NWMR: Melbourne’s North and West Metropolitan Health Region
OoHC: Out-of-home care
OYH: Orygen youth health
RCH: Royal Children’s Hospital
SDQ: Strengths and difficulties questionnaire
TiNT: Tuning into teens
VACCA: Victorian aboriginal child care agency
YSAS: Youth support and advocacy service
